# The critically ill obstetric patient - Recent concepts

**DOI:** 10.4103/0019-5049.71041

**Published:** 2010

**Authors:** Anjan Trikha, PM Singh

**Affiliations:** Department of Anaesthesiology, All India Institute of Medical Sciences, New Delhi - 110029, India

**Keywords:** Critically ill parturient, haemodynamic, intensive care, obstetrics, pregnancy, sepsis, ventilation

## Abstract

Obstetric patients admitted to an Intensive Care Unit (ICU) present a challenge to an intensivist because of normal physiological changes associated with pregnancy and puerperium, the specific medical diseases peculiar to pregnancy and the need to take care of both the mother and the foetus. Most common causes of admission to an ICU for obstetric patients are eclampsia, severe preeclampsia, haemorrhage, congenital and valvular heart disease, septic abortions, severe anemia, cardiomyopathy and non-obstetric sepsis. The purpose of this review is to present the recent concepts in critical care management of obstetric patients with special focus mainly on ventilatory strategies, treatment of shock and nutrition. The details regarding management of individual diseases would not be discussed as these would be beyond the purview of this article. In addition, some specific issues of importance while managing such patients would also be highlighted.

## INTRODUCTION

It is very difficult to conduct well-structured clinical trials in parturients due to which there is a lack of standard guidelines which are available in other critically ill patient populations. Most of the intensivists rely on standard intensive care principles altering them logically considering the physiological changes associated with pregnancy and the need to take care of the foetus. The importance of obstetric ICU is highlighted by the fact that most of these patients are young and disease free initially despite which the mortality still remains higher in comparison to non-obstetric patients. It is important for the ICU team to realize that in a critically ill obstetric patient the safety of the mother is of prime importance, in case one has to choose between the mother and the unborn foetus.

Reasons for ICU admission of obstetric patients can be categorized in to one of the following groups:

1. Conditions related to pregnancy – eclampsia, severe pre-eclampsia, haemorrhage, amniotic fluid embolus, acute fatty liver, and peripartum cardiomyopathy, amniotic fluid embolism, aspiration syndromes, infections etc.
2. Medical diseases that may be aggravated during pregnancy - congenital heart diseases, rheumatic and non-rheumatic valvular diseases, pulmonary hypertension, anemia, renal failure etc.
3. Conditions that are not related to pregnancy – trauma, asthma, diabetes, autoimmune diseases etc.

## INITIAL ASSESSMENT

As for all critically ill patients initial management of such patients consists of a quick history, systemic assessment with an individual organ-based approach with special consideration of the gestational age of the foetus. The treating team should at all times involve the obstetricians as the intensive care team may be ignorant in monitoring the fetal well-being. The viability of the foetus, advantages and disadvantages in continuation of the pregnancy and the mode of delivery, if required are some of the important issues that need to be discussed at the outset. All medications used in an ICU are categorized from A to D (in order of increasing foetal risk) and X (contraindicated); such a list should be readily available for the ICU team. ICU admission criteria for such patients would vary but generally if two organ systems are failing with a need for ventilator support an ICU admission should be mandatory and less sick patients should be cared for in a high-dependency area if available. There are no specific studies regarding various medications used for sedation in this group of patients and depending upon normal ICU practices usual sedatives could be used. It is advisable not to use propofol for sedation in these patients as its risks would outweigh the benefits. The mortality predicting scores like APACHE II/III are not as reliable as in non-obstetric patients; however, the SAPS II score does seem to have some degree of validity in obstetric ICU.[1]

## VENTILATORY STRATEGIES IN A CRITICALLY ILL OBSTETRIC PATIENT

Ventilatory assistance may be required in such a population for varying reasons. Most common causes are - eclampsia, acute respiratory distress syndrome (ARDS; due to aspiration, amniotic fluid embolism etc.) pulmonary oedema, severe cardiac failure, exacerbation of an underlying respiratory disease like bronchial asthma and after major trauma. In addition, many patients with complex cardiac lesions are admitted to an ICU after emergency or elective caesarian sections for supportive ventilation and extubation after haemodynamic stabilization.

Non-invasive ventilation (NIV) has become very popular in the last decade and has shown great benefits in selected group of patients.[2] The status of NIV for obstetric patient is not very promising. Even though it avoids intubation in a difficult and oedematous airway it subjects them to higher chances for aspiration. The patient compliance is also poor due to the need for a tight-fitting mask. It is only advocated to tide over time for a short period provided the patient is fully awake and conscious. Its use has been studied in subset of patients with obstructive airway diseases, and sleep-disordered breathing in pregnancy.[3] If at all a NIV trial has to be instituted it should only be tried in patients who meet the basic pre-requisites required for NIV success – presence of a good respiratory drive, stable haemodynamics and absence of excessive secretions.[4]

All standard protocols need to be adhered to while initiating ventilator support for these patients. Ventilatory strategies for an obstetric patient demand a thorough understanding of the physiological changes in the respiratory parameters during the progress of pregnancy. Natural adaptations for maternal-fetal survival may seem trivial but they form the basis of ventilator adjustments in the intubated pregnant patient. Estrogen mediated airway oedema warrants the use of smaller size endotracheal tubes[5] which increases the resistance to passive expiration. This raised airway resistance may not play a major role inside an operation room but certainly needs a consideration in the ICU setup where prolonged ventilation is needed. It not only increases the oxygen cost of breathing but also becomes a hurdle in successful weaning when spontaneous breathing trials are used.[6] Tracheostomy if required may be difficult due to airway oedema and increased breast size. It is also associated with a greater blood loss due to increased vascularity of the airway.[4]

Not only the oxygen requirements in pregnant patients increase by up to 30-50 ml/min but the closing capacity of the lungs also increases which makes them more liable to desaturate.[7] These patients also need higher PO_2_ values to generate a gradient across the placenta for adequate fetal oxygenation. In contrast to non-obstetric patient where the lower limit of haemoglobin saturation is taken to be around 90% this value for an obstetric patient is close to 95%, which corresponds to a PO_2_ of 70 mm Hg.[8] This not only obviates the need for higher FiO_2_ in these patients but also focuses on the need of ventilatory maneuvers like positive end expiratory pressure (PEEP) which will not only improve the PaO_2_ directly but will also compensate for the increased closing volumes.[4]

Minute ventilation increases by 40% due to progesterone mediated increased sensitivity of respiratory center to CO_2_.[7] This adaptation is clinically important as it lowers the normal PaCO_2_ in pregnant patients nearing term to around 26 mm Hg. It helps to generate a necessary CO_2_ diffusion gradient of around 9 mm Hg across the placenta for foeto-maternal transfer of CO_2_.[9]

These facts alter the optimal ventilator settings in two ways for these patients:

1. Target PaCO_2_ in these patients should be in lower ranges than normal patients to ensure that the foetus is not subjected to acidosis due to hypercarbia.
2. The use of permissive hypercapnea is not advocated in these patients. If used at all, the levels allowed should be kept much lower than what is allowed for non-obstetric patients. However, studies have documented a level up to 60 mm Hg without any fetal compromise.[10]

Although hypercarbia needs to be avoided, respiratory alkalosis too can be detrimental for such patients as it has been associated with a decrease in uterine blood flow.[11] Therefore a PaCO_2_ of about 40 – 45 mm Hg or a pH of about 7.25-7.35 is recommended. There have been reports of falsely lower PaCO_2_ values in pregnant patients when the samples were taken in a supine position, therefore, arterial blood gas sampling should ideally be done in a semi-recumbent position or a sitting position, if possible.[7,12]

The target mechanical ventilation variables need a special mention in this patient population. The pregnancy as such does not alter the lung compliance but the total compliance of the chest wall decreases due to displacement of diaphragm upwards by the gravid uterus.[7] Mechanical ventilation in such patients with poor chest wall compliance can lead to a low trans-alveolar pressure in spite of high airway pressures.[13] Owing to this, the target plateau pressure values may need to be raised from 30 cm H_2_O to achieve acceptable PaCO_2_ and PaO_2_ levels which may not be possible with standard plateau pressure levels.

## SEPSIS AND SHOCK

Obstetric patients commonly are admitted to an ICU with shock which could be due to excessive haemorrhage or due to sepsis or because of a cardiac cause. One of the earliest signs of ongoing haemodynamic compromise is the onset of tachycardia. This, however, may not be such a strong indicator in this subgroup of patients where as a physiological adaptation the basal heart rate is already raised. Sepsis in normal patients presents as unregulated vasodilatation leading to a state of relative hypovolemia but the raised level of progesterone in pregnant females induces a normal vasodilatation. To compensate for this they have a raised blood volume; therefore any septic component is masked by the increased blood volume and is detected only when shock is uncompensated.[14] These haemodynamic changes begin as early as the 8^th^ week of pregnancy and normalize by the 12^th^ week after delivery.[15] After around twenty fourth week the gravid uterus has the potential to compress the inferior vena cava (IVC) and lead to supine hypotension syndrome. The general principles of management of shock in an obstetric patient do not differ much from the rest of the population; however there are few important issues that must be remembered. Since a gravid uterus can cause supine hypotension syndrome the resuscitation of patients in shock should begin with positioning the patient with a tilt to raise the right side.[4] The early goal directed therapy as advocated to treat sepsis in the first 6 hours aims at restoring the decreased perfusion and compensating the oxygen debt due to ongoing anaerobic metabolism. The surrogate markers used for global perfusion continue to be serial measurements of lactate and the Mixed Venous Oxygen Saturation (SvO_2_) even in this subgroup. It is important to realize that near normal values for SvO_2_ may not be adequate for these patients as the normal SvO_2_ in this population in third trimester is around 80% which is significantly higher than the normal population.[16] This finding is probably due to hyper dynamic circulation, increased blood volume and raised absolute haemoglobin mass in these patients. There are however no controlled trials to set a specific target value for the SvO_2_ in this group of patients, so the value of 65% as directed by surviving sepsis campaign still continues to be followed,[17] which may see a change in the future for obstetric patients.

Early resuscitation in sepsis involves administration of a fluid challenge. Fluids need to be administered judiciously in these patients as they are much more liable to develop pulmonary oedema as compared to non-obstetric patients. This may be due to the fact that colloidal oncotic pressure in these patients is lower by around 14 % than normal population, thus pulmonary capillaries may be leakier.[18] Besides this, associated co-morbidities like pre-eclampsia and eclampsia increase predisposition for developing pulmonary oedema. The nature of fluid to be used depends on the clinical scenario. An acutely bleeding patient may more appropriately need blood or a colloid, in patients with risk factors for developing pulmonary oedema colloids may be preferred in view of avoiding larger volume transfusion.

The colloid versus crystalloid controversy persists for obstetric patients as well, as none scores over the other clearly and thus the choice fluid for maintenance should be situation based. It is advised to follow a conservative approach toward total volume of fluids administered. A few authors have advocated even using a negative fluid balance in obstetric patients – the rational being the fact that mortality associated with pulmonary oedema has been found to be much higher than due to acute hypovolaemic renal injury in critically ill pregnant patients.[19,20]

If pharmacological intervention is needed vasodilators like nitroglycerine and hydralazine are fairly safe drugs in pregnancy. There are no specific contraindications to use of furosemide in pregnancy and there are not many human studies to documents its adverse effects on foetus – if any. Of the new drugs which may find their way into list for treating cardiogenic pulmonary oedema in pregnancy include calcium sensitizers like levosimendan[21] and inodilators like milrinone.[22] Levosimendan due to its capability of improving systolic function has been successfully used in peripartum cardiomyopathy to decrease raised pulmonary capillary wedge pressures where conventional agents like dobutamine and dopamine failed to show clinical benefits.[23] Milrinone dilates the pulmonary vessels and increases the cardiac output without tachycardia and hence has a special significance in management of pregnant patients with pulmonary hypertension and low cardiac output states like mitral stenosis.[24]

While choosing a vasopressor in case of severe haemodynamic compromise in such patients the agent’s effect on placental blood flow should be of minimal importance. A severely low blood pressure itself is not capable of perfusing the placenta and in such a situation the only aim should be to raise the blood pressure. In case of associated acidosis it should be kept in mind that all catecholamines may actually be ineffective and vasopressin should be used to support the blood pressure pharmacologically. In conditions associated with septic shock vasoconstrictors like nor adrenalin and vasopressin form agents of choice. In associated low cardiac output states use of dopamine, dobutamine and milrinone is advocated. Epinephrine is a very strong ino-constrictor; however it is important to remember that due to its β 2 agonistic activity it causes uterine relaxation which can delay the progress of labour.

## ANTIBIOTICS

The choice of antibiotic in an obstetric patient needs to give consideration to its safety for the growing foetus and penetration of placenta. The drugs have been classified into groups with respect to their teratogenicity - Group A to D are the antibiotics arranged in order of their suspected teratogenicity and Group X are the ones which are absolutely contraindicated in pregnancy.[25] The safest antibiotics advised to be used as de-escalating therapy in pregnancy are- β lactums, macrolides and aminoglycosides,[26] however one must try to avoid streptomycin as there are reports of fetal ototoxicity with it.[27] The list of drugs that must not be used in pregnancy includes the following – anti-fungal (fluconazole, itraconazole, ketoconazole, griseofulvin), mebendazole, ACE inhibitors, angiotensin receptor blockers, spironolactone, statins and cytotoxic drugs.[28] It is essential to use parenteral antibiotics in all patients who are critically ill, in pregnant obstetric patients this becomes more essential due to the progesterone induced prolonged gastric emptying time[29] which slows the absorption of drugs thus increasing the time to onset of action. Also the increase in pH and mucus production is likely to alter the quantity of drug absorbed – especially weak acidic and basic drugs.[30] As a physiological adaptation the total body water increases by almost 8 liters with blood plasma increasing by almost 50% during pregnancy. This translates into increased volume of distribution affecting more water soluble drugs like β lactum antibiotics,[30] where conventional loading doses cause a lower initial plasma concentration. The above effect may be partially offset by decreased protein binding capacity and thus increasing the free drug concentration even when total actual concentration is lower. However the initial loading doses of antibiotics may actually be logically higher in this subset of patients.

The renal blood flow and glomerular filtration rate increase by 35-60% and 40-50% respectively during pregnancy. As a result the elimination of water soluble drugs primarily eliminated by glomerular filtration also increases by about 50%.[31] Most of the antibiotics excreted via kidney (β lactums etc) are subject to this increased rate of elimination and hence even maintenance doses in obstetric patients need to be on a higher side. Studies have shown a significant lower maximal concentration of ampicillin,[32] imipenam,[33] and first and second generation cephalosporins in such patients.[29] Pharmacokinetics of ceftriaxone, a third generation cephalosporin which undergoes biliary excretion remains almost the same.[34] Thus it is advisable to use the above antibiotics in their higher doses in this patient population.[29] The data with other groups of antibiotics is limited due to their infrequent use in obstetric patients. The treatment of fungal infections in obstetric patients is very challenging as almost all front-line antifungals cannot be used in pregnancy either due to their teratogenicity or due to lack of availability of a safety profile. The only agent considered safe in such case is Amphoterecin B, which automatically becomes the drug of choice for invasive fungal infections.[35] For treating helminthic infections in patients with severe anemia due to worm infestation, the only drug with known safety profile is albendazole; other anti-helminthic drugs are considered unsafe due to known teratogenicity or inadequate studies.

## NUTRITION

As for any other critically ill patient the advantages of enteral nutrition when compared with parenteral nutrition are far too many and it must be the mode of choice whenever possible. A critically ill obstetric patient is more likely to reject enteral feed and develop constipation due to the relaxation effect of progesterone on the bowel smooth muscles. The chances of aspiration can be minimized by ensuring a semi-recumbent position while administering enteral feeds and routine radiological confirmation (if possible) of the position of the nasogastric tube.[4] To combat the reduced motility a prokinetic agent should be added frequently. It is recommended to use anti-aspiration prophylaxis routinely in form of H_2_ blockers or proton pump inhibitors.

The literature available on total parenteral nutrition in an obstetric patient admitted to an ICU is scarce. The target is not only to nurture the critically ill mother but also provide adequate calories and nutrients for the growing foetus. The caloric needs of an obstetric patient can be met with conventionally available preparations with a few modifications. The daily basal requirement of a critically ill patient is around 25 kcal/day/kg (ideal body weight).[36] For an average sized female this turns out to be 2200 to 2800 kcal/day.[37] The specific needs for the foetus vary with each trimester. It is recommended to add around 452 kcal/day in the third trimester and 340 kcal/day in the second trimester. The first trimester usually does not warrant need of extra calories.[38] The protein content of formula feed should be twice that of a non-obstetric patient and this supplementation must be carried on till lactation continues. An optimal nutritional solution needs to add increased amounts of zinc, folate and vitamin B12 in the first trimester.[4] The iron content needs to be almost double that of non-obstetric population, this corresponds to 4-6mg/day.[38] A singleton pregnancy consumes around 1gm of elemental iron.[39] The nutrients that need no special stepping up for preparing formula feeds in obstetric ICU are Vitamin D, E and K as their quantities in regularly used dietary solutions is sufficient to sustain the needs of pregnancy. There is no data available to suggest any alterations in percentages of fats and carbohydrates in critically ill obstetric patients. Thus, lipids should be used to provide 30% of caloric needs and the rest (70%) should come from carbohydrates, proteins in diet should be not be accounted for caloric need as their purpose in ICU is to compensate for negative nitrogen balance.[40] The use of omega 3 fatty acids in diet in obstetric population was advocated to decrease the incidence of preterm labour and pre-eclampsia: although the results of trials are showing no added benefits in this regard.[41,42] Docosahexaenoic acid - a type of omega 3 fatty acid has shown some promise in terms of better outcomes in neonatal visual and neural testing.[43] However more trials are needed to adopt these findings into regular clinical practice.

The nutritional goals must be adjusted on the basis of associated ailments. The target of total calories must be set keeping in mind that as in other critically ill patients use of inotropes may increase caloric requirement by 2.5 times,[44] with each 1 degree Centigrade fever the caloric need increases by 10%[45] and sepsis may increase basal need by 1.9 times.[46,47] The nutritional assessment also is challenging in these patients as weight gain (due to pregnancy associated-gain) and albumin (pregnancy associated decrease) values can not be used as reliable predictors. Indicators like pre-albumin and serum transferrin are preferred for follow up for assessing the response to nutritional support.[48]

## MONITORING

Most patients admitted to an ICU require intensive haemodynamic monitoring and the same is true for obstetric patients. These patients would also need fetal heart monitoring which may not be available as a routine in an ICU. It is advisable to institute invasive monitoring in all haemodynamically unstable obstetric patients.[49] Common indications for this are - pulmonary oedema, severe valvular cardiac disease, cardiomyopathy, embolism, ARDS, eclampsia, persistent oliguria and shock – septic or haemorrhagic. Pulmonary artery catheters have been extensively used in patients with severe cardiac, pulmonary and renal disease but due to an increasing concern about its potential complications non-invasive methods are gaining popularity. It has also been recognized that pulse pressure and stroke volume variation are likely to be better indicators of fluid responsiveness than central pressure and pulmonary wedge pressures.[50] All these have lead to an increased use of non or minimally invasive techniques (transoesophageal echocardiography, Doppler ultrasound, transthoracic bioimpedance and arterial pulse waveform analysis) of cardiac output monitoring in critically ill patients; however there are only a few reports of their usage in critically ill obstetric patients. Doppler echocardiography has been tried in patients with severe PIH and a fair degree of clinical accuracy when compared with pulmonary artery catheters has been reported.[51] However, cardiac output as monitored by thoracic electrical impedance has been reported to be less accurate.[52,53] Pulse wave form analysis based monitors have been found useful in the non-obstetric critical ill population and it is likely that they would be more routinely used in obstetric critically ill patients.

## USE OF FACTOR VII FOR OBSTETRIC HAEMORRHAGE

Activated factor VII has been used in management of postpartum haemorrhage. Presently its use is off label but it has gained popularity in the last few years. It is reported to not only stop active bleeding but has been effective enough to avoid an imminent hysterectomy.[54] In a recent review of its use in pregnant bleeding patients in a dose of 10-70 μg/kg, it was found to also reduce the transfusion requirements.[55] It has also been described to be clinically effective in management of disseminated intravascular coagulation associated with amniotic fluid embolism.[56] It however, needs a stronger clinical evidence to be adopted into routine protocols.

## THROMBOPROPHYLAXIS IN PREGNANCY

The likely hood of developing deep vein thrombosis in obstetric critically ill patients is about four times more than other critically ill patients. This has been attributed to increased secretion of clotting factors due to high estrogen levels and the decreased blood flow in the IVC due to its compression by the gravid uterus.[57] These patients thus must be started on thromboprophylaxis (if there are no other contraindications) as soon as they arrive in the ICU. The drugs that have been studied and recommended are both un-fractionated heparin, and low molecular weight heparins-enoxaparin, dalteparin and tinzaparin.[58] Trials with fondaparinux also have shown it to be efficacious and safe.[59] In case of heparin-induced thrombocytopenia danaparoid has been advocated as the safe alternative.[60] There have been no reports regarding the use of mechanical pumps for DVT prevention in this sub-group of patients but have been effective in other patient populations in an ICU. It is suggested that they should be used as a standard modality for DVT prevention either alone or along with heparins.

## SUMMARY

It is important for ICUs to be prepared to manage critically ill obstetric patients. The most important issue to remember in such cases is that intensivists need to care for two lives. A team approach with an active involvement of the obstetrician is essential. Management strategies regarding mechanical ventilation, nutrition, antibiotic therapy, sepsis management etc need to be suitably modified on the basis of physiological changes seen during pregnancy, perpurieum and the associated medical diseases.
